# Freeze-dried plasma proteins are stable at room temperature for at least 1 year

**DOI:** 10.1186/s12014-017-9170-0

**Published:** 2017-10-27

**Authors:** Jaimie Dufresne, Trung Hoang, Juliet Ajambo, Angelique Florentinus-Mefailoski, Peter Bowden, John Marshall

**Affiliations:** 1 Ryerson University, 350 Victoria Street, Toronto, ON M5B 2K3 Canada; 2 Integrated BioBank of Luxembourg, 6 r. Nicolas-Ernest Barblé, 1210 Luxembourg, Luxembourg

## Abstract

Thirty human EDTA plasma samples from male and female subjects ranging in age from 24 to 74 years were collected on ice, processed ice cold and stored frozen at −80 °C, in liquid nitrogen (LN2), or freeze dried and stored at room temperature in a desiccator (FDRT) or freeze dried and stored at −20 °C for 1 year (FD-20). In a separate experiment, EDTA plasma samples were collected onto ice, processed ice cold and maintained on ice ± protease inhibitors versus incubated at room temperature for up to 96 h. Random and independent sampling by liquid chromatography and tandem mass spectrometry (LC–ESI–MS/MS), as correlated by the MASCOT, OMSSA, X!TANDEM and SEQUEST algorithms, showed that tryptic peptides from complement component 4B (C4B) were rapidly released in plasma at room temperature. Random sampling by LC–ESI–MS/MS showed that peptides from C4B were undetectable on ice, but peptides were cleaved from the mature C4B protein including NGFKSHALQLNNR within as little as 1 h at room temperature. The frequency and intensity of precursors within ± 3 m/z of the C4B peptide NGFKSHALQLNNR was confirmed by automated targeted analysis where the precursors from MS/MS spectra that correlated to the target sequence were analyzed in SQL/R. The C4B preproprotein was processed at the N terminus to release the mature chain that was cleaved on the carboxyl side of the isoprene C2 domain within a polar C terminal sequence of the mature C4B protein, to reveal the thioester reaction site, consistent with LC–ESI–MS/MS and Western blot. Random sampling showed that proteolytic peptides from complement component C4B were rarely observed with long term storage at − 80 °C in a freezer or in liquid nitrogen (LN2), freeze drying with storage at − 20 °C (FD-20 °C) or freeze drying and storage at room temperature (FDRT). Plasma samples maintained at room temperature (RT) showed at least 10-fold to 100-fold greater frequency of peptide correlation to C4B and measured peptide intensity compared to samples on ice for up to 72 h or stored at − 80 °C, LN2, FDRT or FD-20 °C for up to a year.

**Electronic supplementary material:**

The online version of this article (doi:10.1186/s12014-017-9170-0) contains supplementary material, which is available to authorized users.

## Background

The proteins and endogenous peptides of human plasma samples may be purified by partition chromatography with identification and quantification by liquid chromatography, electrospray ionization and tandem mass spectrometry (LC–ESI–MS/MS) [1, 2]. Peptides from blood proteins might facilitate the diagnosis of diseases and the evaluation of the efficacy of therapeutic treatments for individual patients [3–6]. Plasma expresses a weak tryptic protease activity that may slowly degrade the sample proteins over time thus releasing endogenous peptides that may be unrelated to the disease process [7]. The proteolytic activation of the complement system is an important mediator of the acute inflammatory response and humoral immunity [8, 9]. C4B is cleaved to expose a thioester group that permits covalent modification of target macromolecules [10]. It has been shown that levels of C4B peptides may be associated with sampling conditions [2, 11–29]. The steady state balance of ex vivo endo-proteinase and exopeptidase activity may change over time and result in a large variation in the blood peptides observed [2, 30]. Pre-analytical variation in the time the serum or plasma sample remains at room temperature before aliquoting and freezing may be a source of bias in subsequent mass spectrometric measurements [11–24]. The evidence to date indicates that variation in handling immediately after sample collection and prior to centrifugation is the largest source of variation in blood samples [2, 20, 25–27]. To prevent degradation, the sample should be kept on ice during sample handling [31]. Adding serine centered endo-peptidase inhibitors like PMSF or AEBSF [32, 33] to blood fluids in order to preserve the proteins will result in alterations of endogenous peptides [2, 25, 30, 32, 34, 35]. Alternatively, it may be possible to quench ex vivo reactions and store blood samples by freeze drying [3], or rapid drying on filter paper [36] or PVDF [37]. Sensitive and reproducible methods to isolate the cleaved peptides from human plasma have been compared and showed C18 solid phase extraction was a reliable method [38, 39]. C18 solid phase extraction of peptides was used to establish that peptides from C4B are released into plasma at room temperature. Here release of the C4B-peptide (NGFKSHALQLNNRQIR) in human plasma was compared over storage and incubation conditions by random and independent sampling with LC–ESI–MS/MS.

The experiments showed that a plasma sample remaining at room temperature undergoes polypeptide degradation compared to ice cold, frozen samples or freeze dried samples stored at room temperature. Proteolytic degradation of plasma at room temperature resulted in the production of peptides containing the C4B-peptide sequence. Here release of the C4B-peptide (NGFKSHALQLNNRQIR) in human plasma was compared over storage and incubation conditions by random and independent sampling with LC–ESI–MS/MS as confirmed by automated targeted analysis. The C4B peptides showed a low frequency of detection and low ion intensity values in samples collected on ice ± protease inhibitors, but showed a sharp increase at room temperature and remained strongly detectable for days on the laboratory bench. Plasma samples temporarily stored on ice ± protease inhibitors, stored at − 80 °C in or liquid nitrogen, or freeze dried and stored at − 20 °C or at room temperature, all show low levels of C4B-peptide, compared to plasma samples incubated at room temperature for a few hours or days. Here, three methods, random and independent sampling, automatic-targeted quantification, and Western blot all showed that processing of C4B reflects the proteolytic degradation of plasma at room temperature. An additional aim of the experiment was to determine if blood cells are required for the processing of plasma proteins.

## Methods

### Materials

The freeze dryer was from Labconco (Kansas City, MO, USA) and the 30 L pump was from Edwards (Sanborn, NY, USA). The Agilent 1100 HPLC (Santa Clara, CA, USA) for LC–ESI–MS/MS was coupled to an LTQ XL linear ion trap mass spectrometer from the Thermo Electron Corporation (Waltham, MA, USA). The HPLC grade water and acetonitrile were obtained from Caledon Laboratories (Georgetown, Ontario, Canada). The C4B antibody (PA1-9534, Lot No. QB1980891) and the Pierce EZ Link NHS biotinyation kit was obtained from Thermo Fisher Scientific (Waltham, MA, USA). The streptavidin-HRP conjugate was obtained from Jackson ImmunoResearch (West Grove, PA, USA).

### Sample collection

Human plasma was collected under a protocol approved by the Comité National d’Ethique de Recherche (CNER) Protocol #201107 “Biospecimen Research” at the Centre Hospitalier de Luxembourg. EDTA blood samples from 30 male and female healthy subjects, ranging in age from 24 to 74 were collected immediately onto ice, centrifuged at 2000×*g* for 20 min at 4 °C and plasma was aliquoted in 225 μl volumes on ice, and briefly held at − 80 °C prior to randomly assigning to short-term or long-term experimental storage treatments. Short term storage experiments were ice (ICE) or ice plus protease inhibitors (ICE-INH) or room temperature (RT) for up to 96 h as indicated. Long term storage conditions included − 80 °C or liquid nitrogen, (LN2), or freeze drying (FD) followed by storage at − 20 °C (FD-20 °C) or room temperature (FDRT). Freeze dried samples were first frozen to − 80 °C and then rapidly placed in a Labconco rotary sample speedvac with the condenser maintained at − 90 °C with a 30 L per minute pump (a vacuum strong enough to rapidly freeze water) for 24 h before rapidly resealing the vials. There was no heat applied, and no organic solvents or salts were added, and the samples remained frozen under strong vacuum during the drying by means of a − 90 °C condenser and thus were lyophilized, i.e. freeze dried.

### Short term storage experiment

Plasma aliquots (225 µl) from the thirty donors were thawed on ice and then randomly assigned to incubation at room temperature or on ice for varying times. The room temperature (RT) and control samples on ice ± protease inhibitors (ICE and ICE-INH) were incubated for various times, up to 72 h. The protease inhibitors AEBSF, PMSF, benzamidine HCl, and caproic acid were used at 2 mM each with the Sigma Eukaryotic protease inhibitor cocktail at 1/100 (v/v). The Sigma Mammalian Protease inhibitor cocktail contains at least: AEBSF, 104 mM, Aprotinin, 80 μM, Bestatin, 4 mM, E-64, 1.4 mM, Leupeptin, 2 mM, Pepstatin A, 1.5 mM used at 1/100 (v/v). Plasma samples (225 µl) from at least 10 different donors were tested at each time point and over the time course of degradation up to 72 h. At the end of each time period, the samples were frozen, freeze dried and stored dried at − 80 °C until analysis.

### Long term storage experiment

Plasma aliquots from the 30 donors (225 µl) were randomly assigned to a − 80 °C freezer (− 80 °C), liquid nitrogen (LN2), freeze dried and stored in a desiccator at room temperature (FDRT) or freeze dried and stored at −20 °C (FD-20 °C) until analysis.

### Random and independent sampling by LC–ESI–MS/MS

Plasma samples of 25 μl were dissolved in 225 μl of ice cold 5% formic acid prior to collection of the peptides over a preparative, ZipTip, C18 column [39]. The ~ 2 µl elution volume was aspirated and ejected across the C18 resin bed carefully 5 times to avoid permitting air bubbles into the resin bed. Collected peptides were eluted off the ZipTip in 2 µl of acidified 65% acetonitrile and immediately diluted with 18 µl of 5% formic acid and injected for analytical HPLC separation over a 15 cm × 300 µm ID column coupled to an electrospray source for the LTQ XL linear ion trap mass spectrometer (Thermo Electron Corporation). A federated library of human proteins was assembled from NCBI, Ensembl and Swiss-Prot and made non-redundant using Structured Query Language (SQL) [40, 41]. The experimental MS and MS/MS spectra of peptides recorded were correlated to predicted spectra from the federated library at a charge state of 2+ and 3+ to identify fully tryptic peptides using the X!TANDEM [42], OMSSA [43], MASCOT [44] and SEQUEST algorithms [45, 46] set within ± 3 m/z in the precursor mass and within ± 0.5 Da in the fragment mass with up to three missed cleavages [47, 48], as proteins may be only partially digested by proteases. Only the best fit peptide to each MS/MS spectra in terms of charge state or amino acid sequence was accepted.

### Automated targeted LC–ESI–MS/MS

The LC–ESI–MS/MS was repeated with targeted analysis to monitor the C4B-peptide (NGFKSHALQLNNR) released from complement component 4B. The LC–ESI–MS/MS analysis by micro electrospray was repeated but instead of random and independent sampling, the LTQ ion trap was set to monitor NGFKSHALQLNNR [with a monoisotopic mass 1497.78 and so at + 2 charge [M + 2H]^2+^ then (1497.78 + 2)/2 = 749.5 m/z and at + 3 charge [M + 3H]^3+^ then (1497.78 + 3)/3 = 500.26 m/z] with ± 3 m/z. The MS/MS spectra from the targeted LC–MS/MS of the C4B-peptide was confirmed by searching the results against a protein library that only contained the target sequence NGFKSHALQLNNR from C4B using SEQUEST as described above, but with no cut off at 1000 counts, in order to increase sensitivity. The frequency and intensity of precursors within ± 3 m/z of the C4B peptide NGFKSHALQLNNR were confirmed by automated targeted analysis where only the MS/MS spectra that correlated to the target sequence was stored in SQL for Statistical Analysis in R.

### Western blot

A total of 2 µl of EDTA plasma was dissolved in 25 µl of 2 × SDS-PAGE sample-buffer and boiled for 10 min. The EDTA plasma samples were separated over 9% acrylamide discontinuous tris gels with a pH 6.8 stacking gel and a pH 8.8 separating gel in tricine tank buffer at 100 V [49]. The samples were transferred to PVDF (that was pre-wetted in methanol) at 100 V in Towbin buffer [50]. The transfer was confirmed by staining with CBBR in 50% methanol, the position of the molecular weight markers was marked in pencil and the blots erased in pure methanol prior to equilibrating in PBST and Western blot with 1/500 (v/v) biotinylated primary antibody. The binding of the primary antibody was detected with 1/10,000 (v/v) streptavidin HRP conjugate using enhanced chemiluminescence [51].

### Statistical analysis

The precursor ion intensity values that were correlated to C4B tryptic peptides by SEQUEST, together with the parent and fragment m/z and intensity values were automatically parsed into an SQL database [40]. The peptide intensity and frequency values were statistically analyzed using classical statistical approaches such as Chi Square and ANOVA for each protein [2, 52–56]. The peptide intensity values were log_10_ transformed to approximate a normal distribution and then analyzed by ANOVA prior to the Tukey–Kramer Honestly Significant Difference (HSD) and plotted using the R Statistical Analysis System [52, 53, 57]. The Chi Square analysis of the frequency versus intensity histogram for the various treatments was performed and graphed using the generic open-source R statistical system.

## Results

### Random and independent sampling of all peptides from all proteins

**Fig. 1:**
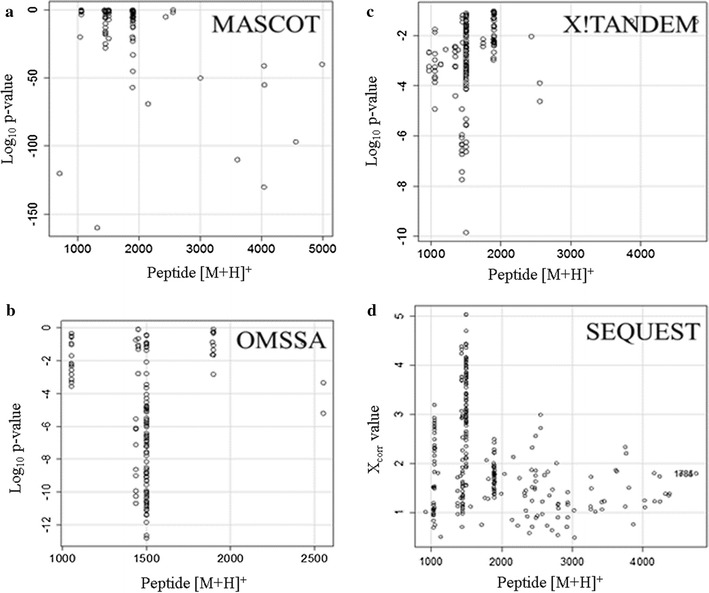
The distribution of *p* values (or X_corr_ values) versus the peptide expected [M + H]^+^ of C4B from the random and independent sampling degraded plasma. The log_10_ expectation values (*p* values) from MASCOT, OMSSA, X!TANDEM and (or X_corr_ values) SEQUEST algorithms are plotted against peptide [M + H]^+^ values. **a** MASCOT a heuristic probability-based MOWSE score algorithm; **b** OMSSA a heuristic probability algorithm; **c** X!TANDEM a goodness of fit algorithm; **d** SEQUEST a modified Pearson cross correlation algorithm (X_corr_). C4B was the most commonly observed protein by many independent peptides in room temperature samples

**Fig. 2:**
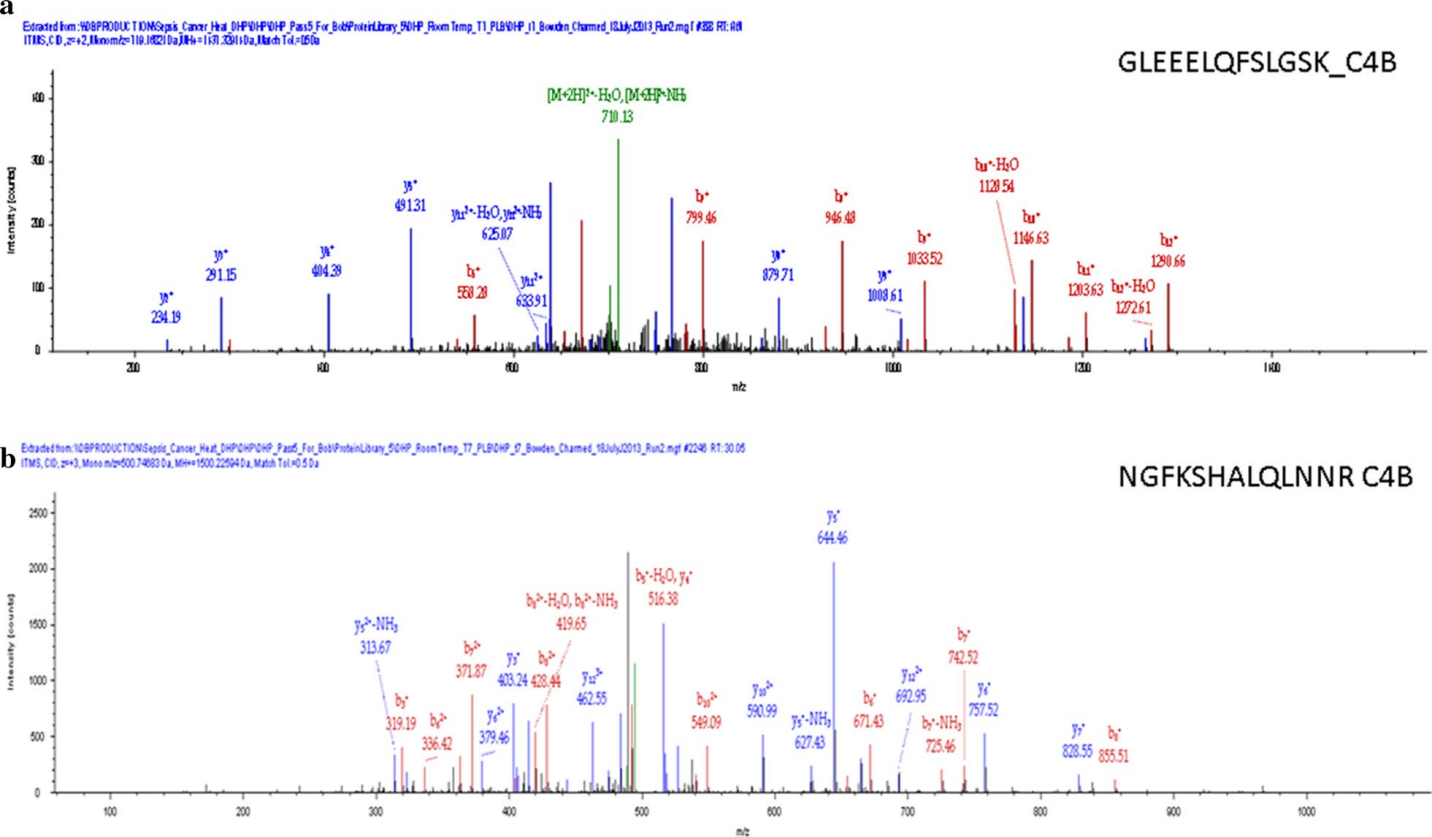
Typical examples of the MS/MS spectra of unique peptides from complement C4B. **a** MS/MS spectra that correlated to the fully tryptic amino acid sequence GLEEELQFSLGSK with [M + H]^+^ 1437.33 and a z value of 2+; **b** MS/MS spectra that correlated to the fully tryptic amino acid sequence NGFKSHALQLNNR with [M + H]^+^ 1500.23 and a z value of 3+. The MS/MS fragment ions are labelled exactly as provided by the Proteome Discoverer system that used the SEQUEST algorithm provided by Thermo Fisher. The MS/MS spectra were obtained from random and independent sampling of all precursor ions during unbiased LC–ESI–MS/MS

**Fig. 3:**
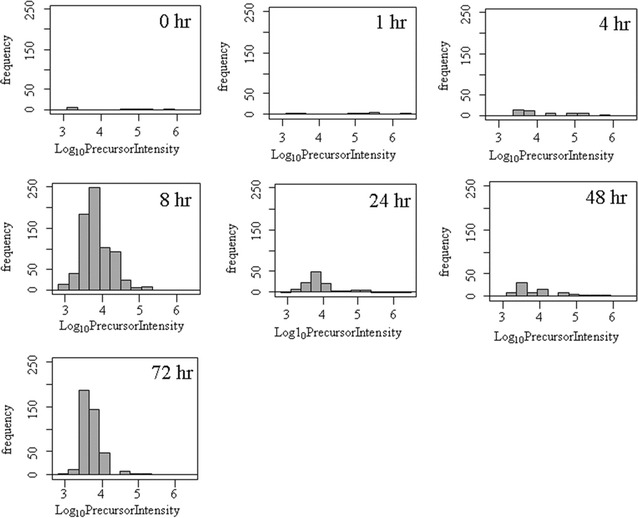
The histogram of log_10_ precursor intensity of the C4B-peptide (NGFKSHALQLNNR) by random and independent sampling over incubation of EDTA plasma on ice versus room temperature over time. The precursor ion intensity filter of 1000 counts was imposed before examining the precursor with the fitting algorithms. Random and independent sampling showed a 10-fold to 100-fold increase in C4B-peptide detection with incubation at room temperature

**Fig. 4:**
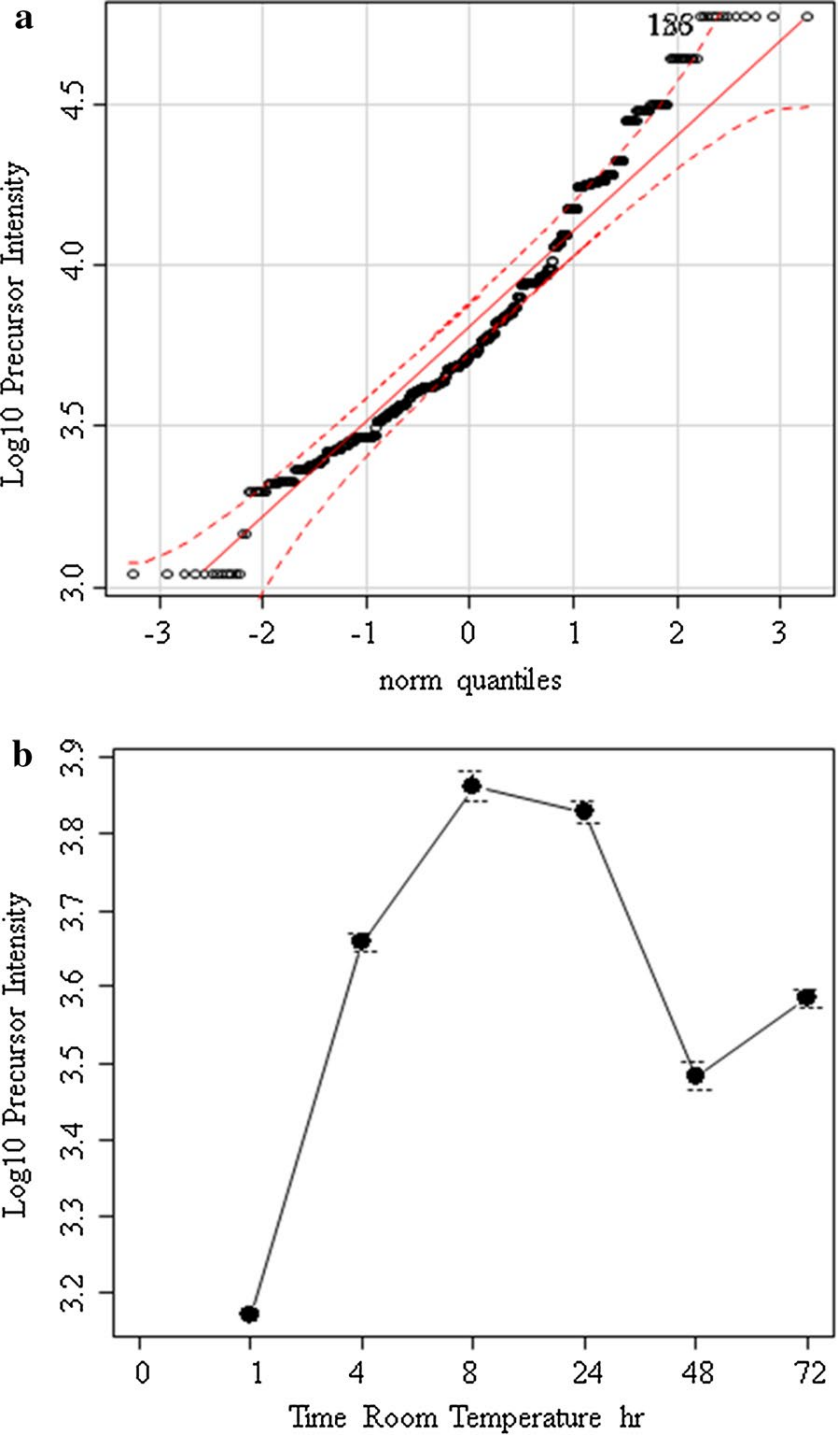
The quantification of C4B-peptide (NGFKSHALQLNNR) by random and independent sampling by LC–ESI–MS/MS. Panels:** a**, the quantile plot of the randomly sampled C4B peptides;** b**, the intensity values of the randomly sampled C4B-peptide log_10_ intensity value (arbitrary counts) over time. The probability that the time points were the same by ANOVA was *p* ≤ 0.001. The time point 0 refers to samples maintained on ice

The tryptic peptides in human EDTA plasma were analyzed by random and independent sampling of the 5 highest intensity peptides eluting at any moment from the HPLC–ESI–MS/MS system. The MS/MS spectra were correlated to the tryptic peptides of the human proteome in a federated protein library with up to three missed cleavages that revealed the processing of complement component 4B (C4B) (Table 1, Fig. 1). Random and independent sampling of C4B from ice cold or room temperature EDTA plasma by LC–ESI–MS/MS with correlation by MASCOT, OMSSA, X!TANDEM and SEQUEST showed *p* values for individual MS/MS spectra that ranged from p < 0.1 (E-1) to p < E − 150 (Fig. 1). Plasma samples that were maintained on ice over time showed very little cleavage of C4B in contrast to samples incubated at room temperature that showed the cleavage of the C4B peptides (Table 2, Fig. 2). The C4B peptides NGFKSHALQLNNR and GLEEELQFSLGSK were among the most commonly observed in agreement with previous results [29]. The release of peptides from complement C4B was readily detected in room temperature samples by unbiased LC–ESI–MS/MS (Table 2, Fig. 2). The C4B peptides were almost undetectable at time zero (ICE), but were clearly detectable in as little as 1 h or more at room temperature. After incubation at room temperature for 4–8 h the most characteristic peptide from C4B (NGFKSHALQLNNR) was observed with greater frequency that was apparently significant by the Chi Square test (Fig. 3) that indicated a low probability (< 0.0001) that the room temperature samples were the same as the control. The intensity distribution of the C4B-peptide approached normality as assessed by quantile plots (Fig. 4a). The C4B-peptide intensity values were typically less than 1000 intensity counts at time zero (ICE). The C4B-peptide showed detectable intensity values after 1 h with a mean of just above 1000 intensity counts, that increased by almost an order of magnitude over 4–8 h at room temperature (RT).

**Table 1:** The peptide counts to complement C4B protein sequences from correlation analysis of the MS/MS peptides detected in human plasma

Total count	Filter	Gene symbol	MASCOT	OMSSA	X!TANDEM	SEQUEST
972	0	*C4B*	145	248	311	268
857	1	*C4B*	145	238	308	166
583	2	*C4B*	145	119	155	164
		Sum	E-2610	E-2492	E-1129	X_corr_ 1192

The MS/MS spectra were correlated to charge (z) of 2+ and 3+ with up to three missed cleavages of fully tryptic peptides with precursor ions within ±3 m/z and MS/MS fragments matched within 0.5 Da. The data were filtered by possible charge states (Filter 1) and amino acid sequences (Filter 2) to ensure that only the single best fit of the MS/MS spectra was accepted. The sum *p* value or X_corr_ (a function of cross correlation) value for filter 2 is listed. The cumulative *p* values for all C4B data with filter 2 ranged from E-2492 from OMSSA, E-1129 for X!TANDEM and E-2610 from MASCOT (where E-2 is considered significant) while SEQUEST showed a sum X_Corr_ of 1192 (where 2.5 to 3.75 is considered significant). The sum total peptide correlation counts from preserved samples, control samples and time course samples from both random sampling and target analysis in this study

**Table 2:** The endogenous peptides of C4B detected by LC–ESI–MS/MS of samples where any peptide correlation to C4B by X!TANDEM was accepted

Peptide sequence	Log10 mean intensity	STND ERR	N
EELQFSLGSK	3.59	0.03	4
GFKSHALQLNNR	4.22	0.00	2
GLEEELQFSLGSK	3.59	0.19	18
GLEEELQFSLGSKINVK	3.66	0.38	12
GLEEELQFSLGSKINVKVGGNSK	3.56	0.00	2
HALQLNNR	3.70	0.06	2
NGFKSHALQLNN	3.85	0.22	14
NGFKSHALQLNNR	3.79	0.32	156
NGFKSHALQLNNRQI	3.78	0.09	6
NGFKSHALQLNNRQIR	3.79	0.26	56
QFSLGSKINVK	2.99	0.00	2
SHALQLNNR	3.72	0.19	20
SHALQLNNRQIR	3.87	0.18	10
STQDTVIALDALSAYWIASHTTEERGLNVTLSSTGR	5.52	0.00	2
TLEIPGNSDPNMIPDGDFNSYVR	3.53	0.11	4
VTASDPLDTLGSEGALSPGGVASLLRLPRGCGEQTMIYLAPTLAASR	4.78	0.00	1

The most commonly detected discrete peptide sequences were NGFKSHALQLNNR and GLEEELQFSLGSK

### Automatic-targeted LC–ESI–MS/MS analysis

**Fig. 5:**
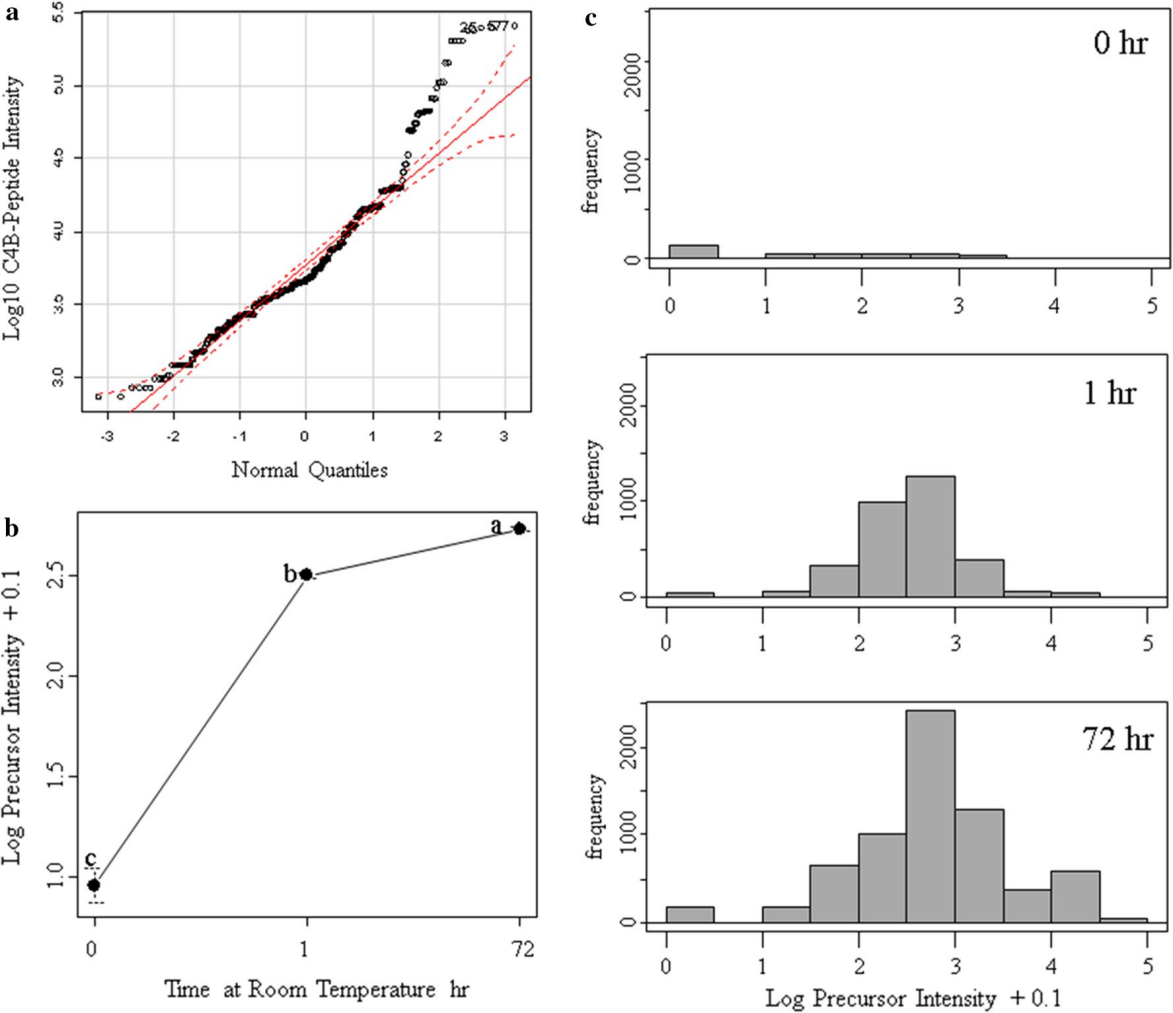
The automatic-targeted analysis of the complement C4B-peptide (NGFKSHALQLNNR) at time 0, 1 and 72 h incubation at room temperature. **a**, The normality of the log_10_ transformed intensity data as assessed by quantile plot; **b** the mean and standard deviation of the targeted precursor intensity value (different letters indicate a significant difference by the Tukey–Kramer Honestly Significant Difference Test); **c** the histogram showing the frequency of observing peptides in each log_10_ intensity bin. Automatic targeted sampling showed a 100-fold increase in frequency and about a 10-fold to 100-fold increase in C4B-peptide intensity with incubation at room temperature

Random and independent sampling showed a large increase in C4B detection frequency and intensity in plasma with time at room temperature but has greater sampling error compared to targeted measurement of C4B peptides. The MS/MS spectra from peptides with precursors within ± 3 m/z of the predicted 2^+^ or 3^+^ m/z values were automatically searched against the C4B-peptide NGFKSHALQLNNR using the SEQUEST algorithm. The correlated peptide count and precursor intensity values were automatically collected in SQL Server for the automatic transformation, computation of means, normality, intensity differences by ANOVA and frequency differences by Chi Square using the R statistical analysis system (Fig. 5). The fully automated targeted analysis of the C4B peptide NGFKSHALQLNNR using SQL and R showed a log_10_ intensity distribution of the peptide that approached Gaussian normality (Fig. 5a). Comparing baseline plasma samples versus those incubated for 1 or 72 h at RT confirmed a sharp increase in average peptide intensity values from time 0 to 1 or 72 h at room temperature by ANOVA followed by the Tukey–Kramer test (Fig. 5b). The NGFKSHALQLNNR peptide counts from all precursors increased dramatically from time 0 (on ice) to thousands or even tens of thousands of counts by as little as 1 h and up to 72 h at room temperature (Fig. 5c).

### Primary structural analysis of C4B

**Fig. 6:**
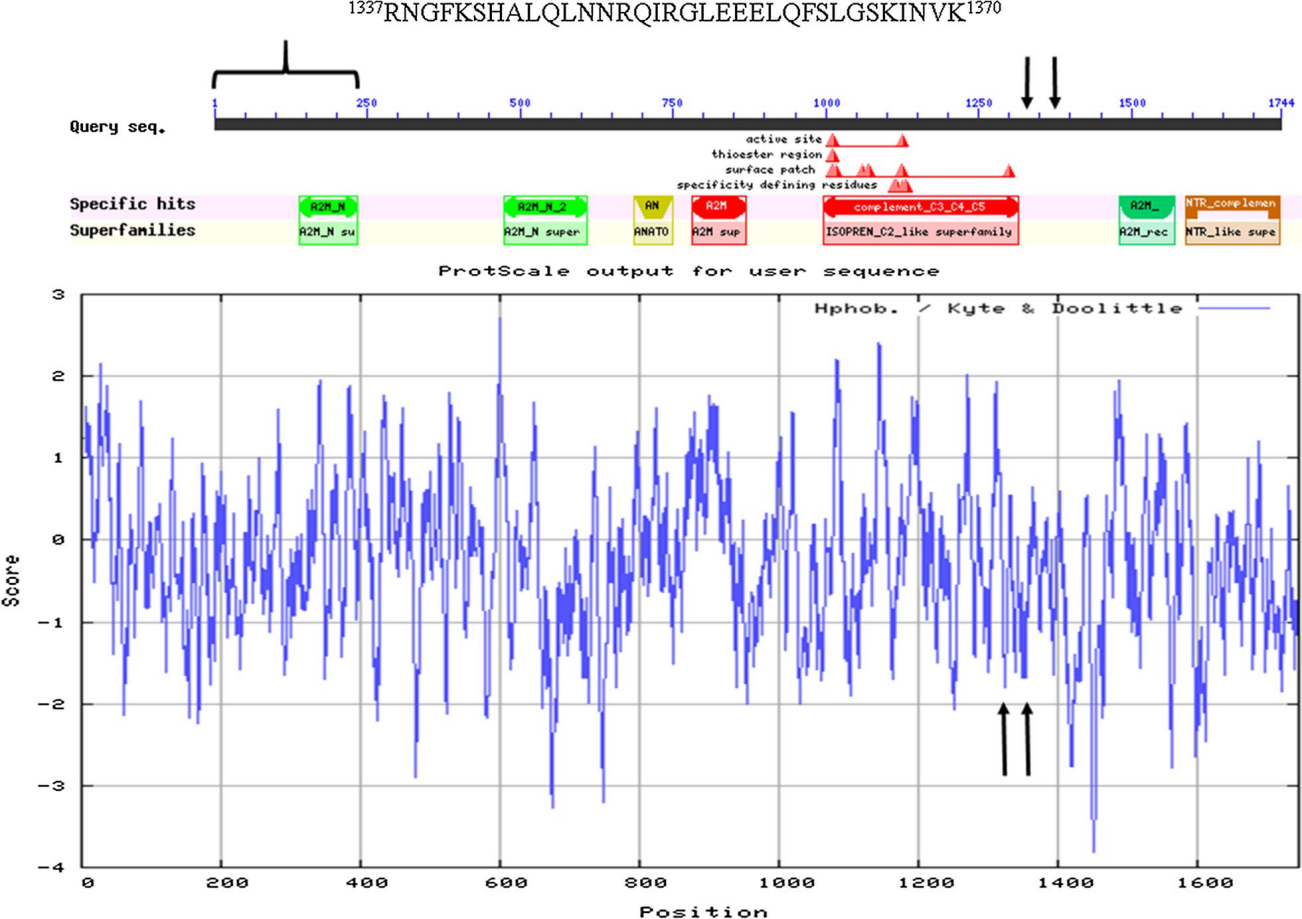
The location of the most common peptide sequences that were cleaved within the complement C4B protein from the protein accession NP_001002029.3 The peptide sequence NGFKSHALQLNNRQIR shows no significant relationship to any other protein and contained 31% of all observed peptides from C4B. The peptide sequence GLEEELQFSLGSKINVK accounted for 11% of all peptides. The arrows show the locations of the main peptide cleavage sites on the carboxyl side of the isoprene C2 domain at a site within a sequence of basic, acid and polar amino acids. The bracket shows a section of the preproprotein that is also cleaved upon warming to room temperature producing the mature chain detectable by Western blot. The C4B amino acid sequence ^1337^RNGFKSHALQLNNRQIRGLEEELQFSLGSKINVK^1370^ on the carboxyl side of the isoprene C2-like superfamily domain was the most frequent site of cleavage (see arrows). The sub-sequence ^1137^NGFKSHALQLNNR^1352^ is located just to the carboxyl terminal side of a local hydrophilic maximum and flanks the evolutionarily conserved isoprene C2 sequence shared by the innate defense proteins complement 4A/B and alpha 2 macroglobulin. The cleavage sites of the peptide(s) NGFKSHALQLNNRQIR are flanked on the amino side by short stretches of hydrophilic amino acids, the released peptide includes asparagine and glutamine, and there is a stretch of three glutamic acid residues and glutamine adjacent on the carboxyl side of the cleavage site

Peptides consistent with the cleavage of the C4B preproprotein to yield the mature chain were observed (Fig. 6). The most commonly detected cleavage site on the mature C4B chain was on the carboxyl side of the isoprene C2 domain shared with alpha 2 macroglobulin as reflected by LC–ESI–MS/MS. A frequent C4B cleavage site was within the polar C terminal sequence ^1337^RNGFKSHALQLNNRQIRGLEEELQFSLGSKINVK^1370^ (NP_001002029.3) that contained the most commonly observed peptides NGFKSHALQLNNR and GLEEELQFSLGSK (Fig. 6).

### Western blot against the mature C4B protein

**Fig. 7:**
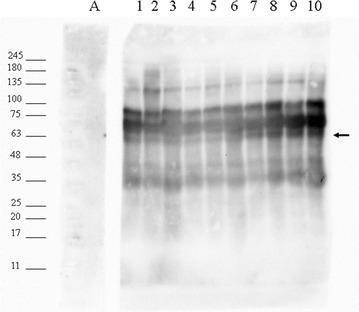
Western blot against the mature C4B protein chain in human EDTA plasma over the time course of degradation at room temperature. The monoclonal antibody against C4B was biotinylated and detected with Streptavidin-HRP. The arrow indicates the expected C4B peptide after proteolysis. Lanes: MW, Molecular weight marker as indicated; A, Streptavidin HRP alone; 1, time 0; 2, 1 h; 3, 1 h; 4, 4 h; 5, 8 h; 6, 24 h; 7, 36 h; 8, 48 h; 9, 72, hr; 10, 96 h. Molecular weight markers for a 9% Tricine SDS-PAGE gel are shown in kilodaltons (kDa). The Western blot analysis showed the increased formation of mature C4B with time and the development of an additional band with time at room temperature confirming the proteolytic processing of C4B. Complement C4 is expressed as a high molecular mass preproprotein that reacts poorly with the antibody raised against the mature protein that exits in blood as a 79 kDa mature chain in close agreement with the most intense band in the Western blot that appears roughly in line with the 75 kDa molecular mass marker. Proteolytic cleavage of the C-terminal domain from C4B starting at NGFKSHALQLNNR to the carboxyl terminus of the C4B protein should yield a 63 kDa protein (see arrow). Control blots stained with Coomassie Blue confirmed equal loading of all lanes (see Additional file 1: Fig. S1). These data confirm the finding shown in Table 2 and Fig. 2. The manufacturer (THERMO) indicates the antibody recognizes an 88, 75 and 33 kDa form of C4B in good agreement

Samples of EDTA plasma were resolved by tricine SDS-PAGE and blotted to PVDF supports for Western analysis. The Western blot showed that the antibody recognizes the processed form of mature C4B as expected. Western analysis strongly detected the parent C4B protein in close agreement with the predicted relative mass of 84,163 Da and its major cleavage product at 71,539 kD based on the annotation found at NCBI NP_001002029.3 (Fig. 7). The cleavage of the C-terminal portion of the C4B chain should yield a protein of about 63 kDa as observed (Fig. 7).

### Plasma sample long-term storage by freezing and freeze-drying

**Fig. 8:**
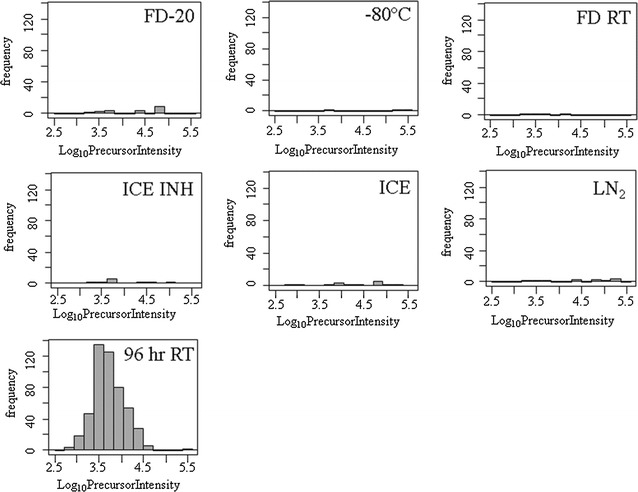
The histogram showing a comparison of the preservation methods of ice ± protease inhibitors, freezing versus freeze drying alongside a positive control for sample degradation as illustrated by C4B-peptide (NGFKSHALQLNNR) by random and independent sampling by LC–ESI–MS/MS. Storage treatments: freeze drying and storage at room temperature (FDRT); freeze drying and storage at −20 (FD-20); freezing storage at −80 °C (−80); or liquid nitrogen (LN2); versus plasma degraded for 96 h at room temperature (96 h RT). The histogram shows the frequency of peptides in each log_10_ intensity bin. The precursor ion intensity filter of 1000 counts was imposed before examining the data in R

Plasma was protected from degradation, as measured by the release of C4B-peptide, by freeze drying followed by 1 year storage at room temperature (FDRT), freeze drying followed by 1 year storage at − 20 °C (FD-20 °C), 1 year freezing at − 80 °C or 1 year freezing in liquid nitrogen (LN_2_) (Fig. 8). Furthermore, there were no signs of proteolytic degradation after short-term storage of plasma samples on ice ± protease inhibitors, for up to 3 days (Fig. 8). The Chi Square test showed a low probability (< 0.0001) that the degraded samples were the same as the control.

## Discussion

This study regarded the cleavage of the C4B protein at room temperature compared to ice cold, frozen, or freeze dried plasma samples by random and independent sampling, or automatic targeted quantification by LC–ESI–MS/MS, and SDS-PAGE with Western blot. All three analytical methods show a similar trend of little detectable C4B-peptide cleavage in ice cold, frozen or freeze dried plasma samples. Cleavage of C4B rapidly became apparent after incubation for 1–4 h at room temperature using sensitive LC–ESI–MS/MS.

### Random and independent sampling

Without any pre-conceived notion of what proteins or peptides might be the best for measuring sample degradation, random and independent sampling was used to compare all possible human tryptic peptides across all sample storage conditions. The counting of peptides to proteins across treatments with SQL/R was a simple means to identify the proteins in human plasma that degrade with incubation at room temperature. The C4B-peptide levels may serve as a marker of sample degradation based on the ≥ tenfold increase in the frequency of detection or peptide intensity over time at room temperature. We conclude that the use of random and independent sampling of peptide frequency and/or intensity values may serve as a means to quantify the cleavage of C4B. In randomly and independently sampled experiments the intensity values at time zero for the C4B-peptide NGFKSHALQLNNR were apparently below the noise cut off used in this statistical experiment but the peptide was detectable by 1 h at room temperature and showed a marked increase in both frequency and intensity by 8 h. The large differences in the peptides frequency and intensity over time indicated that the cleavage of the peptide NGFKSHALQLNNR from complement C4B may serve as a measure of the degradation of the sample due to endopeptidase activity at room temperature after sample collection [1, 2]. Random and independent sampling using unbiased LC–ESI–MS/MS is a costly and time consuming approach that is necessary to make unbiased discoveries of candidate markers but is not an efficient means to assay an individual protein.

### Automatic-targeted analysis of Complement 4B NGFKSHALQLNNR

Automatic targeted analysis showed that the C4B-peptide (NGFKSHALQLNNR) was readily detected in the ice control and degraded plasma samples and increased by about ≥ tenfold by 1 h at room temperature. After log_10_ transformation, the C4B-peptide intensity was Gaussian and could be used to provide relative quantification and statistical analysis using ANOVA. The automatic-targeted quantification of the precursor peptide ion of the C4B-peptide is a promising step towards creating a quantitative assay for the quality control of EDTA plasma samples. In this approach a low resolution, yet robust and sensitive, ion trap is set to monitor the precursor window where automatic computations based on the fit of the MS/MS spectra to the target peptide sequence resolves the intended precursor from any ions with similar m/z values.

### Complementary sampling strategy

Random and independent sampling from a totally random experimental design is the statistical gold standard for avoiding false discovery. Pooling representative samples, or taking ratios of isotopic or isobaric tags may reduce or eliminate measured biological and sampling error leading to false discovery. Moreover, making a ratio of isotopic or isobaric peptides, such as ICAT ratios, lacks independence and tends to multiply the error in the two samples (denominator error × numerator error) leading to reduced statistical power. Hence the random and independent sampling of separate biological samples is an appropriate strategy to discover real differences between populations that provides a realistic assessment of population variance. In a complementary approach, the automatic targeted method captured biological variation but avoided sampling error by a high frequency sampling of the targeted analyte that shows that C4B intensity levels increased within 1 h at room temperature and remained elevated and increased for at least 72 h that showed little sampling error. Only the intensity and frequency values of precursors within ± 3 m/z that yielded MS/MS spectra automatically correlated to the target peptide by the SEQUEST algorithm were accepted in SQL. Thus the fit of the MS/MS spectra ensured the validity of the precursors accepted into the dataset for automated statistical analysis in R. The combination of random and independent sampling, together with automatic targeted confirmation, as demonstrated for the first time here, is a sensitive and practical approach to biomarker discovery in human plasma that avoids false discovery and yet automatically provides confirmatory measurements with low technical error.

### Room temperature versus ice, frozen or freeze-dried samples

Storing samples on ice was an effective means to prevent proteolytic degradation for up to 3 days. The use of C4B-peptide counting and precursor intensity versus frequency analysis clearly indicated that freeze drying and storage at room temperature, or freeze drying and storage at − 20 °C were just as effective in preventing sample degradation as freezing at − 80 °C or liquid nitrogen for up to 1 year. However degradation of complement C4B commences very rapidly upon thawing and does not reflect the stability of most plasma proteins and so unless the samples are collected on ice, freeze dried and reconstituted on ice ± protease inhibitors some degradation of C4B is likely unavoidable.

## Conclusion

Three different methods, random and independent sampling, automatic-targeted analysis, and Western blot all agreed that the cleavage of C4B gives a good indication of the sample incubation at RT. The time that a clinical plasma sample remains at room temperature was a key factor in the cleavage of C4B. The simplest explanation of the observations here is that a tryptic protease activity in human plasma acts ex vivo at room temperature to cleave a proportion of the total pool of complement C4B and this processing may be directly detectable by sensitive LC–ESI–MS/MS or immunological methods. We conclude that the complement proteins, that are part of a proteolytic cascade, are especially sensitive to ex vivo degradation of EDTA plasma during sample warming. The peptide NGFKSHALQLNNR from complement 4B showed a rapid increase in the frequency and intensity of detection after warming to room temperature and increased with hours of incubation and then remained elevated over the subsequent days. Thus, after all blood cells are removed from plasma on ice, C4B peptide levels remained low, but yet subsequently increased with time at room temperature entirely in the absence of white blood cells. All of the results are consistent with the expression of endogenous complement fragments that represents a balance of endoproteinase activity versus exopeptidase activity. The C4B peptide assays indicates that the highly soluble proteins in human plasma may be preserved by freeze drying and entirely reconstituted upon the addition of water and so freeze drying is an attractive option for sample preservation that would also permit robust and reliable shipment of samples at low cost.

## Additional file

**Additional file 1: Fig. S1.** A replicate CBBR stained gel to confirm the equal loading of the Western blot shown in Figure 8 of the main paper. (see legend of Figure 8 for details).

## Abbreviations

AEBSF: 4-benzenesulfonyl fluoride hydrochloride
ACN: acetonitrile
C4B: complement component 4B
C4B-peptide: NGFKSHALQLNNR
ICE: fresh EDTA plasma stored on wet ice
ICE_INHIB: fresh EDTA plasma stored on wet ice with protease inhibitors
RT: fresh EDTA plasma stored at room temperature
LN_2_: fresh EDTA plasma stored in liquid nitrogen
FD: freeze dried EDTA plasma
FD-20 °C: freeze dried EDTA plasma stored in an electric freezer at − 20 °C
FDRT: EDTA plasma freeze dried and stored in a desiccator at room temperature
PMSF: phenylmethane sulfonyl fluoride
PVDF: polyvinylidene fluoride
