# Supplementary material for: Freeze-dried plasma proteins are stable at room temperature for at least 1 year
Source: Clin Proteomics. 2017 Oct 27;14:35. doi: 10.1186/s12014-017-9170-0 (PMC5659006; doi:10.1186/s12014-017-9170-0)
Supplement: Supplementary file 1 — Additional file 1: Fig. S1. A replicate CBBR stained gel to confirm the equal loading of the Western blot shown in Figure 8 of the main paper. (see legend of Figure 8 for details). [file 12014_2017_9170_MOESM1_ESM.docx]

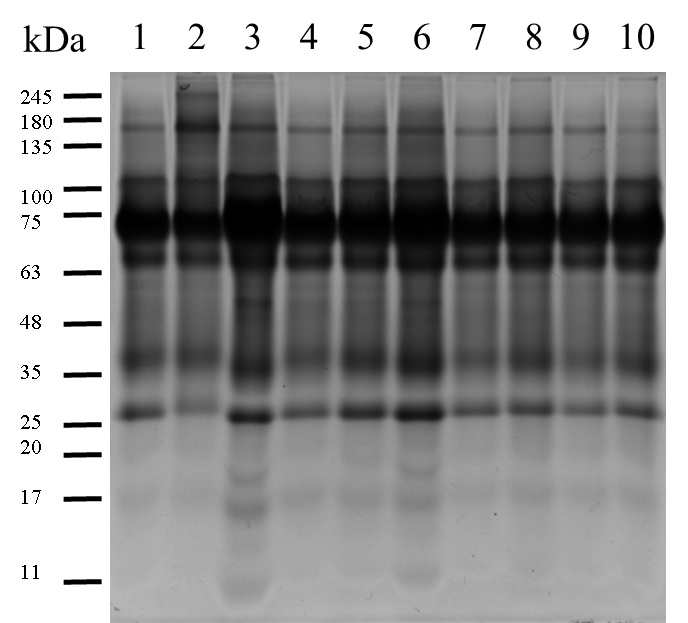


Additional file 1: Fig. S1. A replicate CBBR stained gel to confirm the equal loading of the Western blot shown in Figure 8 of the main paper. (see legend of Figure 8 for details).
